# Efficacy of Neoadjuvant Cemiplimab Treatment for Cutaneous Squamous Cell Carcinoma—A Systematic Review

**DOI:** 10.3390/ijms26168109

**Published:** 2025-08-21

**Authors:** Maria Eduarda Palomba, Julia Adriana Karmirski, Flávio Carneiro Hojaij

**Affiliations:** 1 Faculdade de Ciências Médicas da Santa Casa de São Paulo, FCMSCSP, Sao Paulo 01224-001, SP, Brazil; 2 Faculdade de Medicina FMUSP, Universidade de Sao Paulo, Sao Paulo 05508-220, SP, Brazil

**Keywords:** Cemiplimab, neoadjuvant, efficacy, cutaneous squamous cell carcinoma, immunotherapy, PDL1 inhibitor

## Abstract

Skin cancer is the most common cancer form worldwide, and it is primarily divided into melanoma and non-melanoma types, with non-melanoma being the most prevalent condition. Cutaneous squamous cell carcinoma (cSCC) accounts for 50% of primary skin cancers and is characterized by uncontrolled keratinocyte proliferation. cSCC’s current standard treatment is surgical resection and chemotherapy. Unfortunately, these methods often lead to disfigurement, functional morbiditly, and compromised function. In contrast to immunotherapy, emerging scenarios have shown promising results, especially in neoadjuvant settings. Cemiplimab (Libtayo^®^; Regeneron, Tarrytown, NY, USA), a PD-1 monoclonal antibody, has shown efficacy in treating advanced or metastatic cSCC, and its use as a neoadjuvant therapy has been recently explored. This review aims to evaluate Cemiplimab in the neoadjuvant setting for cSCC treatment. The Methodology followed PRISMA guidelines, this review analyzed studies on Cemiplimab as a neoadjuvant therapy for cSCC that were sourced from PubMed, Web of Science, and Scopus. Only controlled trials, cohort studies, case series, and systematic reviews were included. From 341 records, 21 studies were included, and six clinical trials provided key data about neoadjuvant Cemiplimab’s response rates, efficacy, adverse effects, and safety considerations. The targeted data revealed a neoadjuvant Cemiplimab mean pathologic response rate of 72%, with a 62% objective response rate. Treatment-related adverse events (TRAEs) affect 66% of patients, though most cases are not severe. The most common include fatigue, maculopapular rash, and diarrhea. The studies showed high rates of complete pathological responses (cPRs) and major pathological responses (mPRs), suggesting a strong therapeutic potential. Neoadjuvant Cemiplimab for cSCC therapy shows high response rates, low recurrence, improved survival, and manageable side effects. The current literature indicates that Cemiplimab may also be effective when used in immunosuppressed patients. Despite more research still being needed to confirm its long-term benefits and the effects of the drug’s use outside of clinical trials, there is strong evidence to consider neoadjuvant Cemiplimab as a promising and efficient treatment.

## 1. Introduction

Skin cancer is the most common cancer type worldwide. It can be categorized into melanoma and non-melanoma subtypes, with the latter one being more common. There are two types of non-melanoma skin malignancies: squamous cell carcinoma (SCC) and basal cell carcinoma (BCC) [1]. The worldwide SCC incidence increases each year, having an important impact on the economy, life quality, morbidity, and mortality [2].

Cutaneous squamous cell carcinoma (cSCC) accounts for 50% of primary skin cancers, and it is caused by an abnormal and uncontrolled proliferation of keratinocytes [3]. cSCC is the world’s second most common skin cancer [4]. It is characterized by a high tumor mutational burden, and the most frequent clinical signs are patches, plaques, and tumors [2,3]. The standard treatment for cSCC is the surgical excision and nonsurgical therapies if complete resection is not possible [5,6,7]. Although it is not a current widespread standard of care, immunotherapy has been showing a positive impact in cSCC resection, even more so when associated as a neoadjuvant treatment [8,9,10]. An immunotherapy alternative is Cemiplimab (Libtayo^®^; Regeneron, Tarrytown, NY, USA), which is a PD-1 receptor monoclonal antibody (human IgG4 antibody) that binds to PDL1 receptor [9]. The overexpression of PDL1 may be a mechanism of resistance in tumor cells, enabling evasion of the immune system through PD-1/PDL-1 binding [10]. Moreover, specifically when considering cSCC, one of its immunogenicity patterns is the upregulation of immune checkpoint molecules (PD-1 and PD-L1), which also suggests the efficacy of immunotherapy treatment [10,11].

PD-1 is an inhibitory receptor present in T cells, and its physiological function is the maintenance of peripheral tolerance. The interaction of this receptor with the ligands PD-L1/PD-L2 limits T cell proliferation, cytokine production, and cytotoxicity, avoiding ineffective and exacerbated immunological responses. These ligands are normally expressed in macrophage and mesenchymal, dendritic, B, stem, and other non-hematopoietic cells [12,13]. Considering some cancers’ PDL1 overexpression mechanism of resistance, immune checkpoint inhibitors, such as Nivolumab, Pembrolizumab, and Cemiplimab, were developed. These drugs improve antitumor responses by T cells while also reducing peripheral tolerance. However, this reduction may cause some treatment-related adverse effects, such as skin rash events, fatigue, and gastrointestinal issues [10,14].

Currently, the use of Cemiplimab is approved by the Food and Drug Administration (FDA) for the treatment of patients diagnosed with non-small cell lung cancer, for patients with metastatic or locally advanced cSCC, and for BCC patients with locally advanced or metastatic basal cell carcinoma [15]. In Brazil, the National Health Surveillance Agency (ANVISA), also recommends the use of this medicine for similar uses [16]. Cemiplimab applications for carcinoma are not limited to squamous cell types but haves also been showing efficacy for treating other epithelial malignancies.

Cemiplimab, either as a monotherapy or a chemotherapy adjuvant, has shown efficacy as treatment of advanced non-small cell lung cancer, providing improved survival outcomes and leading to regulatory approval in this setting [17,18]. In the treatment of recurrent or metastatic cervical cancer, Cemiplimab improved the overall survival and had high objective response rates, which led to the FDA approval for this medicine. There are still ongoing trials and studies, aiming to establish Cemiplimab’s efficacy for other HPV- related cancers (vaginal, vulvar, and anal carcinomas) [19]. Moreover, in basal cell carcinoma (BCC), Cemiplimab has demonstrated clinical relevance, and it is FDA-approved for patients with locally advanced or metastatic BCC [20,21].

Recently, the use of this monoclonal antibody has been explored as a neoadjuvant treatment. Pilot studies have been showing high percentages of complete pathological response after treatment with Cemiplimab as a neoadjuvant therapy. Despite the evidence from the studies mentioned before, Cemiplimab is not widely used in clinical practice as a neoadjuvant treatment. In this context, this review focuses on evaluating the efficacy of Cemiplimab in neoadjuvant settings for cSCC, also emphasizing this treatment’s implications and safety, and aiming for better clinical guidance and knowledge.

This review provides strong insights into the efficacy of Cemiplimab in the neoadjuvant setting as a promising treatment for cSCC, which contributes to advancing the knowledge and guidance in this field. We believe that the manuscript will be of interest to your readers due to its relevance and significant social, economic, and health contributions.

## 2. Materials and Methods

This review was conducted following the PRISMA (Preferred Reporting Item for Systematic Reviews and Meta-Analyses) guidelines. All the reported data were obtained from the available published literature, so institutional review board approval and informed consent were not required. The review protocol was registered on PROSPERO (CRD420250650512).

### 2.1. Inclusion and Exclusion Criteria

This systematic review aimed to gather scientific articles on the neoadjuvant use of Cemiplimab in patients with cutaneous SCC.

The strategy used for elaborating this review’s question was the PICO tool, which consists of the population (P), patients diagnosed with cutaneous squamous cell carcinoma; intervention (I), treatment with Cemiplimab in a neoadjuvant; comparison (C), none; outcomes (O), the course of the patient after the treatment; study type (S), controlled trials, retrospective cohort studies, systematic reviews, and case series. Studies were excluded if (a) Cemiplimab was not used as a neoadjuvant settings treatment for cSCC; (b) data of patients after the treatment were not extractable; (c) the study reported fewer than five patients; (d) the article type was a conference abstract, case report, or book chapter; or (e) cSCC data presented could not be separated from other tumor types. No restriction on the publication date was applied.

### 2.2. Data Source and Study Search

An electronic search strategy was performed on each of the following databases: Web of Science (Clarivate, Philadelphia, PA, USA) PubMed (National Library of Medicine, Bethesda, MD, USA), and Scopus (Elsevier, Amsterdam, The Netherlands) on 12 December 2024. The search strategy employed was as follows: “Cemiplimab AND (neoadjuvant OR neoadjunvacy) AND (‘basosquamous carcinoma’ OR ‘squamous cell carcinoma’ OR ‘squamous cell cancer’ OR ‘cutaneous squamous cell cancer’).”

### 2.3. Selection of Studies and Data Extraction

Sources in the form of letters, reports, or formal studies that reported on primary cutaneous SCC treated with Cemiplimab as a neoadjuvant were included. In total, 28 articles were included after a full-text review by two independent reviewers (J.K., M.E.P.) Duplicates were removed using the systematic review management platform Rayyan systematic review software (Qatar Computing Research Institute, Hamad Bin Khalifa University, Doha, Qatar). To evaluate the eligibility and the articles’ relevance, titles, abstracts, and full texts were screened. Discrepancy resolution and verification from the selected articles were executed by the senior author (F.H.). Data were archived in an Excel Version 2.99.25080110 (Microsoft Corp., Seattle, WA, USA) spreadsheet. Data collected from the articles included neoadjuvant Cemiplimab response rates, Cemiplimab dosing, efficacy outcomes, study-related adverse effects frequency, Cemiplimab efficacy and safety considerations, and treatment groups limitations. The complete pathologic responses (cPR)—absence of viable tumor (living tumor cells) in the post-treatment surgical specimens—rates data were extracted from the articles, as well as the major pathologic responses (mPRs)—≤10% of viable tumor in the post-treatment surgical specimens. To ensure the scientific rigor of this systematic review, the PRISMA (Preferred Reporting Items for Systematic Reviews and Meta-Analyses) statement and checklist were used.

### 2.4. Risk of Bias and Study Quality Assessment

Two separate authors assessed the methodological quality of the included studies independently. Since none of the included cinical trials were randomized trials, the Methodological Index for Nonrandomized Studies (MINORS) criteria were used to measure the study quality.

## 3. Results

### 3.1. Electronic Database Search Results

**Figure 1:**
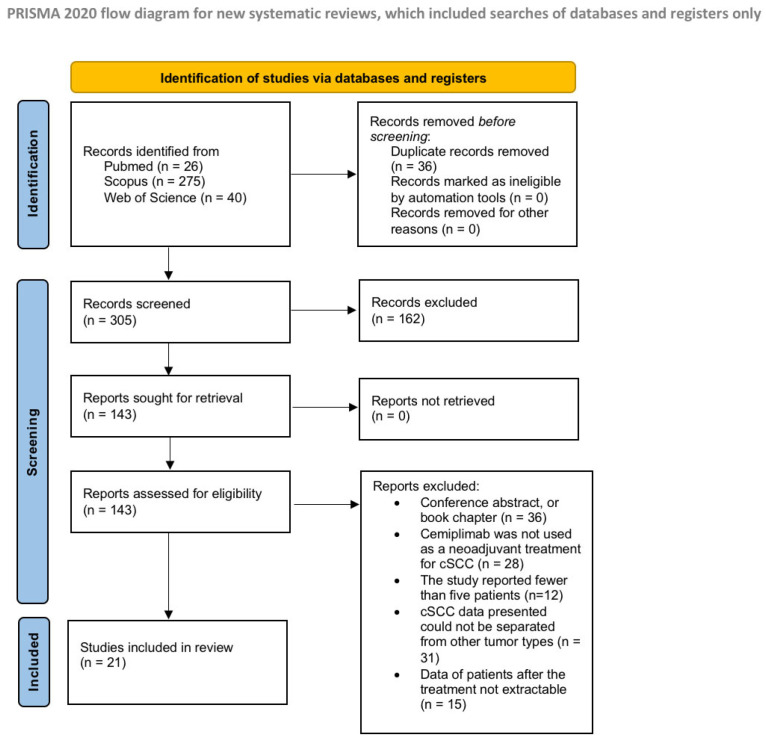
Preferred Reporting Items for Systematic Reviews and Meta-Analysis flow diagram (PRISMA).

A total of 341 records were identified from the preliminary search. Before screening, 36 duplicates were removed, and after the title and abstract screens, 143 articles were sought for retrieval. As a result of the application of the inclusion and exclusion criteria, 21 articles were included in the review [11,12,20,21,22,23,24,25,26,27,28,29,30,31,32,33,34,35,36,37]. A flow chart of the study justifying the exclusion reasons and inclusion process is shown in Figure 1.

### 3.2. General Features of the Reviewed Clinical Trial

In this review, the included studies were selected based on their high relevance to the topic under investigation. Among the 21 studies, 17 consist solely of previously published literature, primarily literature reviews. However, three clinical trials and one case series had been completed as of the present date and are therefore discussed in greater detail in Section 3.3 and Section 3.4. GTwo other currently on-going linical trials’ data are also discussed in Section 3.3 and Section 3.4, despite not being considered as part of the included studies number since they are still ongoing. This review excluded studies in the format of conference abstracts, so the ongoing Ascierto et al. NEO-CESQ study [34] and Wong et al. pilot study [3] could not be included in this study. Both datasets were presented at the American Society of Clinical Oncology (ASCO) 2023 annual meeting. A total of 158 neoadjuvant Cemiplimab treatments will be performed for resectable stages II–IV CSCC patients (AJCC-8). The patients’ mean pathologic response rate, which included a complete pathologic response (absence of viable tumor in the post-treatment surgical specimens) or major pathological response (≤10% viable tumor in the post-treatment surgical specimens), was 72%. The mean objective response rate was 62%. The patients’ mean treatment-related adverse events (TRAEs) were fatigue, maculopapular rash, and diarrhea. The mean rate of patients who presented any TRAE was 66%. The studies’ efficacy data and TRAEs are presented in Table 1 and Table 2.

### 3.3. Risk of Bias Assessment

Among the total of 21 included studies, 7 were nonrandomized studies, which means they did not use any randomization strategies when assigning participants to treatment groups. Out of the seven nonrandomized studies, scores ranged from 8 to 14. The most frequent deficiencies encountered in the included studies were a lack of prospective calculation on the study size and excessive loss of patients, which did not allow follow-up. All the studies adequately reported a clear aim and an unbiased assessment of the study endpoints. The MINORS scores for the included studies are listed in Appendix A (see Table A1, which displays the MINORS scores of the included studies).

### 3.4. Phase II Trial Data

Six active clinical trials are examining the neoadjuvant treatment of Cemiplimab. From these studies, Ferraroto et al. [25] and Gross et al. [19], both phase II trials, showed similar pathological responses, considering both complete and major responses (Table 1). In the Ferrarotto et al. 2021 pilot phase II trial, 20 patients with stages II–IVA cSCC received neoadjuvant Cemiplimab [14]. In total, 11 of these patients had a complete pathologic response (cPR), and 3 of them had a major pathologic response (mPR). The 12-month outcomes data showed a 95% disease-specific survival (DSS), 89% disease-free survival (DFS), and 95% overall survival [24]. None of the patients who presented a pathological response (cPR or mPR) had a recurrence, and there were no treatment-related fatal events (Ferraroto et al., 2021) [24]. Seven patients experienced treatment-related adverse events (TRAEs), all of which were fully resolved. The most common symptoms reported were pruritus and a maculopapular rash (Table 2). Another phase 2 nonrandomized study (Gross et al., 2022) [26] was conducted with stages II–IVA cSCC (AJCC-8) patients who received four 350 mg neoadjuvant Cemiplimab IV each before resection. Out of the 79 enrolled patients, 70% had some reduction in viable tumor (living tumor cells) in the post-treatment surgical specimens (51% cPRs; 13% mPRs). Also, from this latter study, none of the responders had recurrence, and there was only one patient death suspected to be treatment related. TRAEs occurred in 57 patients, presenting fatigue, maculopapular rash, and diarrhea; 3 of these patients had grade 3 immune-related events (Table 2). The one- and two-year post-surgery follow-ups showed favorable outcomes: 89% 1-year event-free survival (EFS), 85% 2-year EFS, and 92% 1-year DFS. In light of the biomarker analyses conducted by Rischin et al. [23], an increased clonal abundance and enhanced immunological response throughout T cells was noted in the patients of this study (Gross et al., 2022) [23,26]. Th Gross et al. [14] and Ferraroto et al. [25] studies concluded that the treatment is a promising option for treating cSCC, considering the high response rate and outcomes; additionally, no new safety signals for Cemiplimab were identified in a neoadjuvant setting.

Some studies included high-risk cSCC patients, whose data may differ from those that do not include them, especially considering the response rates and outcome results. Emily Y. Kim et al. [29] performed a relatively small cohort study that evaluated 27 patients with advanced stages I—IV cSCC (AJCC-8). Different from most clinical trials, 33.3% of the patients in the data presented had a concomitant diagnosis of lymphoma. A third of their treatment group would have been excluded from prior neoadjuvant Cemiplimab clinical trials, leading to differences in the reposted results. The overall pathologic response reported was 47.4% (Table 1), which is lower than the rate reported by Ferraroto et al., 2021 [24,25], and Gross et al., 2022, in their trials [14,26]. This study’s 1-year patient outcomes data were an 83.3% progression-free survival rate, 91.7% DSS, and the patient’s recurrence-free survival rate was 90.9%. Only one of the responders had a recurrence. Overall, Emily Y. Kim et al.’s 2024 study supported the previous literature, considering the neoadjuvant Cemiplimab efficacy, but also highlighted the lower responses when considering higher-risk patients [29].

### 3.5. Phase I Trial Data

A phase I clinical trial data from a NEO-CESQ study was presented at the 2023 ASCO annual meeting [37]. There were 23 high-risk stages III/IVCSCC-HN (AJCC-8) patients enrolled in this phase 1 single-arm trial. They received two cycles of neoadjuvant Cemiplimab in total, 47% of these patients had a cPR or mPR pathologic response (Table 1), and 29 patients had TRAEs (Table 2). Moreover, activity, data, and results are awaited. Wong et al. also presented the data of another ongoing pilot study at ASCO 2023 [38]. The recruited patients include I–IV surgically resectable cSCCs, and the treatment setting consists of cetuximab loading dose with neoadjuvant Cemiplimab followed by three cycles of chemotherapy (cisplatin or carboplatin + docetaxel) with cetuximab and Cemiplimab prior to definitive surgery. Out of the 10 already enrolled patients, there was a 100% pathologic response, 40% cPRs, and 60% mPRs (Table 1). Out of the adverse events (Table 2), the most common were rash, nausea, fatigue, and diarrhea; one patient experienced severity grade 3, and another one grade 4 (Wong et al., 2023) [38]. In 2024, 20 new patients were enrolled in this same study, and the data continues to show a high response rate (Dunn et al., 2024) [40].

### 3.6. Case Series Data

A case series presented by Goldfarb et al. [28] also included some high-risk patients. Out of the six enrolled patients affected with primary CSCC-HN stages II–IV (AJCC-8), only four were able to complete the treatment and undergo periorbital resection. All of them had some pathologic response, where 50% had cPRs and 50% mPRs [28] (Table 1).

Through the next Section 3.4, Section 3.5 and Section 3.6, the six clinical trials (four from the included studies [14,25,28,29,37,38] and two still ongoing [3,39]) are discussed. They were grouped by considering whether they were phase I or II trials and case series.

## 4. Discussion

Cutaneous Squamous cell carcinoma (cSCC) is the world’s second most common skin cancer. Despite usually having a favorable prognosis, 5% of the patients can develop an advanced cSCC stage [6]. Patients who present locally advanced forms and metastasis have a poor prognosis, with an 89% 5-year mortality due to distant metastasis and a 2-year decreased expected median survival [7,33,35]. The standard treatment is surgical intervention, but for unresectable situations, irradiation is a possibility. Systemic therapies can be part of the treatment strategy when surgery or chemotherapy is not possible in situations of advanced or distant metastatic disease [20]. Commonly, the applied systemic therapies are platinum-based cytotoxic agents and agents targeting the epidermal growth factor receptor (EGFR) [31]. These traditional methods often lead to disfigurement, functional morbidity, compromised function, limited efficacy, poor tolerability, and potential toxicity. As such, there is an urgent need for safe alternative therapeutic strategies to enhance cosmetic results and patients’ quality of life (QoL), providing long-lasting response rates [22,33]. Therefore, different studies have been exploring the use of Cemiplimab in a neoadjuvant setting for cSCC patients.

Cemiplimab in the neoadjuvant setting is an emergent and promising treatment for cSCC patients, especially when considering the presented data from recent clinical trials. The average pathologic response rate data extracted from the evaluated studies was 72%. Gross et al.’s phase 2 study reported a 70% pathologic response rate (pCRs and mPCRs) with neoadjuvant anti-PD-1 therapy in solid tumors, which is, to date, the highest rate for current neoadjuvant anti-PD-1 therapy results in solid tumors, highlighting Cemiplimab’s efficacy. Also, within the data extracted from 158 trials, there was only one fatal event, which could have possibly been treatment-related (Gross et al., 2022) [26]. Therefore, considering the different treatment outcomes from the included literature, the neoadjuvant Cemiplimab setting presents high pathologic responses, low treatment-related discontinuation rate, and rare severe study-related adverse effects, thus supporting its efficacy.

### 4.1. Immune Implications, Safety, and Tolerability

Considering the sSCC immunogenicity pattern, and given the high tumor mutational burden, immune checkpoint inhibitors indicate a promising alternative for treating cSCC [11,37]. Given this fact, immunotherapy has been explored for sSCC patients, and Cemiplimab has already been FDA-approved for locally advanced and metastatic forms [15]. This drug in adjuvant and neoadjuvant settings has presented rapid and durable responses, favorable survival, and well-tolerated toxicities in the majority of patients [32]. Under these considerations, Cemiplimab was referred to by the Italian Association of Medical Oncology as “[…] a curative approach for a disease that lacked a clean standard of care in its advanced stage” [7]. Another emergent alternative for the current challenging advanced cSCC clinical scenario is neoadjuvant immunotherapy, which already demonstrates favorable pathological responses and positive long-term outcomes. In most included reviews and clinical trials, neoadjuvant Cemiplimab treatment is well tolerated, with no serious adverse events occurring after the treatment [41]. Then, the neoadjuvant use of Cemiplimab represents a compelling alternative for sSCC patients.

Specifically, when exploring the neoadjuvant setting, studies have shown an even higher pathological complete response frequency, cost-effectiveness, and QoL improvement when compared with the standard treatment settings [22]. The included articles show that the use of Cemiplimab in a neoadjuvant setting leads to less invasive surgeries, with better cosmetic and function-preserving outcomes [24,25,29]. Also, the use of Cemiplimab is feasible and successful for de-escalation strategies [22,26]. Neoadjuvant therapy, when compared with adjuvant therapy alone, allows earlier identification of response and survival biomarkers, and achieves a broader immune response, as shown by a greater expansion and diversity of anti-tumor T cells [21]. Immune checkpoint inhibitor (ICI) adjuvant approaches can cause immunological homeostasis disruption by reactivating cellular immunity, resulting in dysfunctions and other treatment-related adverse events (TRAEs). However, neoadjuvant therapy with ICIs did not demonstrate this correlation. They showed, instead, a superiority of this setting in safeguarding outcomes [22]. Other absent phenomena in adjuvant immunotherapy are the neoadjuvant ICIs’ capacity to form effective immune memory to multiple antigens, thereby preventing postoperative immune escape. It also enhances systemic anti-tumor immunity that targets and eliminates distant micrometastases and increases the role of non-hematopoietic cells [12,21]. Furthermore, this treatment approach opens the opportunity for better and earlier analysis of the post-neoadjuvant tumor specimen. This allows for the refinement of long-term clinical outcomes prediction and better guidance of post-surgical therapies to improve patients’ post-treatment life quality. As such, the Cemiplimab in a neoadjuvant setting could contribute to less invasive and disfiguring resections, lower recurrence, better survival and QoL outcomes, and enhanced tumor-specific immune responses. 

ICI treatment can affect the immune system signaling and biomarkers, responses, mechanisms, and molecular pathways in many ways. Examples of immune modulator effects can be enhanced T cell activation, enhanced T cell tumor infiltration, and decreased Myeloid Derived Suppressor Cells (MDSCs) and Tregs within the tumoral microenvironment. A phase 2 clinical trial (Gross et al., 2022) [26] revealed an inflamed tumor immune microenvironment when analyzing pretreatment tumor biological specimens of patients who achieved a pathological response after neoadjuvant Cemiplimab therapy for resectable cSCC [23,26]. This suggests that these patients may have memory CD8+ T cells, as shows by CD45RO and EOMES expressions as drivers of a complete tumor regression [23,24,25]. The 2-year follow-up data from this trial showed increased expression of effector T-cell-related genes and enrichment of T cell activation, the interferon-g/a response, and TCR signaling pathways [23]. Most of this data contrasts with the immune scenario that patients with no pathological response presented. These enhanced systemic activations of tumor-specific and non-specific T cells are also demonstrated by another pilot phase II study (Ferrarotto et al., 2021) [24]. Better activation of the systemic immune response was observed since the checkpoint blockade before surgery yielded more antigen-specific T cells. Overall, the studies show a systemic anti-tumor immunity increase, which positively affects surgical resection, lowers recurrence, and increases survival [12,22].

When considering the treatment-related adverse effects, a skin rash was the third most common one. As reported by Gross et al. [26], Ferraroto et al. [24], and Wong et al. [41], 20.2% of the 109 patients presented wih a maculopapular rash. The dermatologic effects were predominantly mild to moderate, with grade 1 or 2 severity, and there was no need for treatment discontinuation. Unfortunately, the studies did not provide details about the severity or extent of the skin rash events. Moreover, peripheral blood eosinophilia does not appear to be an adverse reaction associated with Cemiplimab, either in the neoadjuvant setting or as a first-line or adjuvant therapy [24,26,34,36,41].

### 4.2. Suitable Treatment Candidates

Immunocompromised patients (human immunodeficiency virus (HIV), hematologic malignancies, advanced solid organ malignancies, solid organ or hematopoietic stem cell transplantations, autoimmune conditions) are considered at high risk of developing the advanced form of cSCC. Unfortunately, many of the actual studies do not include immunocompromised patients in their trials due to safety considerations and the high rejection rate [30]. Recipients of solid organ transplants (SOTs) face a risk of developing cSCC that is up to 250 times higher than that of the general population [35,36,37]. It may be challenging for them to be included in trials considering the increased T cell activation after ICI treatment, possibly leading to allograft rejection [32,36]. These treatment group exclusion criteria can be considered a barrier to real-world neoadjuvant Cemiplimab efficacy [13,42]. Another patient group that is also commonly excluded is those diagnosed with hematological malignancies, not only because of rejection rates but also for presenting lower responses to the treatment [29]. Overall, about 30 to 40% of all patients have benefited from ICIs [22]. Given this information, the identification of suitable candidates is necessary [41].

### 4.3. Neoadjuvant Immunotherapy Treatment Considerations

In the context of neoadjuvant immunotherapy treatment (NAIT), it is essential to highlight key information for analyzing the effectiveness of neoadjuvant Cemiplimab. Despite the high responsiveness and the reduced surgical resection, the residual tumor’s boundary and surroundings can be obscured because of the treatment-related associated adhesion, fibrosis, immune cell infiltration, and an inflammatory environment [25,29]. In addition to the referred obscurement, some of NAIT’s response patterns are responsible for compromising imaging techniques, the predictive biomarkers examination, and the tumor re-biopsy, leading to complications at subsequent surgical interventions [9,20,22].

### 4.4. Therapeutic Implications of Neoadjuvant Cemiplimab in cSCC Patients

Neoadjuvant use of Cemiplimab is emerging as a promising and potentially practice-changing strategy in the management of resectable cutaneous squamous cell carcinoma (cSCC), particularly in patients at high risk of recurrence or surgical morbidity. Including phase II trials and retrospective case series [14,25,29], Cemiplimab has demonstrated substantial pathological response rates, with pathological complete responses (pCRs) reported in 30% to over 50% of patients, and additional major pathological responses in a significant proportion [14,25,28]. Although patient cohorts have varied, ranging from individuals with large or nodal disease to those with smaller tumors in functionally or cosmetically sensitive locations (like the periorbital region [28]), consistent tumor regression has been observed across all these groups. This broad applicability suggests Cemiplimab’s potential to reduce tumor burden preoperatively, facilitate margin-negative resections, and allows more conservative surgery, implying better cosmetic and QOL outcomes.

From a safety perspective, Cemiplimab has been well tolerated in the neoadjuvant setting. Immune-related adverse events such as fatigue, rash, and diarrhea have been the most reported, with grade ≥ 3 toxicities occurring infrequently and manageable. This shows Cemiplimab’s superiority in relation to traditional chemoradiation regimens, which often carry higher toxicity burdens [24,26,37,38,41]. These findings demonstrate wide therapeutic implications: in addition to surgical downstaging and morbidity reduction, the early systemic activation of anti-tumor immunity offers the potential to target micrometastatic disease, possibly improving long-term outcomes. These effects are even pronounced in high-risk populations, also demonstrating similar pathologic response rates and adverse effects’ severity and manageability. However, further studies are needed to clarify the benefits in lower-risk or less advanced cases [20,29].

Even though there are still some variabilities to cover, neoadjuvant Cemiplimab’s demonstrated efficacy and manageable safety profile support its incorporation for the neoadjuvant setting treatment for cSCC patients. The therapeutic implications of neoadjuvant Cemiplimab in resectable cSCC include effective tumor downstaging, reduced morbidity, lower recurrence, and improved long-term cosmetic and QoL outcomes. These findings support its consideration as a valuable alternative to the actual standard cSCC treatments.

### 4.5. Limitations and Future Directions

The main limitations of this study encompass the lack of patients with severe comorbidities, immunosuppression, and secondary neoplasia present in the included studies. Given this fact, an analysis of the efficacy, safety, and tolerability may be limited [30]. Nevertheless, some real-world setting studies and case series are revealing that elderly and immunosuppressed patients may exhibit pathological and clinical responses similar to those seen in patients from clinical trials with specific inclusion criteria [32,39]. Regarding the real-world setting safety, tolerability data have also been comparable with clinical trials, indicating neoadjuvant Cemipimab is a feasible treatment, even in the immunosuppressed, the elderly, and patients with multiple comorbidities [41,42,43].

Although current studies have explored various aspects of Cemiplimab in the neoadjuvant treatment of cutaneous squamous cell carcinoma (SCC), certain variables are still underexamined. No study evaluated the histological grade of squamous cell carcinoma as a factor in assessing the response to neoadjuvant therapy. This represents a possible limitation since it would be of extreme importance for studies that correlate the histological grade of carcinomas considering the therapy response and highlighting its role.

The systemic therapies (platinum base and EGFR) most amplie to cSCC [20,38] typically present recurrence, limited responses, and early progression, in contrast to Cemiplimab, where fatal adverse events are rare, with high response rates and being safe and well tolerated. Further studies are needed in order to confirm the long-term toxicity profile in terms of efficacy and safety in real-world setting treatment groups [30,32,36].

Considering the present trials, none have assessed the histological grade as a potential predictor of treatment response. This omission stands out as a limitation and highlights the need for future research that incorporates this to better elucidate Cemiplimab’s therapeutic role.

## 5. Conclusions

In conclusion, neoadjuvant Cemiplimab therapy for cSCC patients shows high response rates, tolerability and safety, lower recurrence, and improved survival. Fatal adverse events are rare, and TRAEs are immune-mediated and usually well managed. Although future studies are necessary to analyze its feasibility in real-world settings, some case series already indicate comparable results between the current trials and immunosuppressed patients. Finally, the benefits seem to outweigh the risks, and it is considered a promising and efficient treatment.

## Figures and Tables

**Table 1:** Efficacy data (pathologic and imaging response rates) from neoadjuvant Cemiplimabt reatment of cutaneous squamous cell carcinoma clinical trials.

Authors	Treatment Groups	Cemiplimab Setting	CPR ^1^	MPR ^2^	NR + PPR	OR
Gross et al. [14]	Resectable AJCC-8 stages II (at least 3 cm), III, or IV (M0) CSCC (n = 79)	350 mg IV every 3 weeks for up to four doses before resection	51%	13%	25%	68%
Ferraroto et al. [25]	Primary or recurrent resectable CSCC-HN stages III–IV (AJCC-8) (n = 20)	350 mg IV every 3 weeks for 2 cycles before resection	55%	15%	30%	30%
NEO-CESQ [33]	Resectable AJCC-8, high-risk stages III/IV (MO) CSCC-HN (n = 23)	350 mg every 3 weeks for 2 cycles before resection	39%	8%	5% (PPR) 48% (NR)	-
Kim et al. [29]	Resectable CSCC stages I–IV (AJCC-8) (Cemiplimab; n = 22) (Pembrolizumab; n = 5)	Cemiplimab—350 mg every 3 weeks for 2 to 4 cycles before resection Pembrolizumab—200 mg every 3 weeks or 400 mg every 6 weeks	36.8%	10.5%	52.6% (PPR) 0.1% (NR)	50%
Wong et al. [38]	Participants with locally advanced, resectable CSCC-HN stages T1, N2-3; T2, N1-3; and T3/T4a, any N (AJCC, 8th ed.) without evidence of distant metastasis (M0) (n = 10)	Cetuximab loading dose with 350 mg Cemiplimab followed by 3 cycles of chemotherapy (cisplatin or carboplatin + docetaxel) with cetuximab and Cemiplimab prior to definitive surgical resection	40%	60%	0%	-
Goldfarb et al. [28]	Primary CSCC-HN stages II–IV (AJCC-8) (n = 6)	2 injections of Cemiplimab 3 weeks apart before resection	50%	50%	-	100%

^1^ cPR—complete pathological response. ^2^ mPR—major pathological response.

**Table 2:** Most common types of adverse effects from neoadjuvant Cemiplimab treatment of cutaneous squamous cell carcinoma clinical trials.

Types of Treatment-Related Adverse Effects	
Frequency ^1^	
Any event	66%
Fatigue	25%
Diarrhea	13.7%
Nausea	12.8%
Maculopapular rash	20.2%
Pruritus	11.9%
Severity ^2^	
Treatment-related discontinuation	1.5%
≥Grade 3	4.5%
Study-related death	0.75%

^1^ Adverse effects frequency data was extracted from the following articles: Gross et al. [18], Ferraroto et al. [24], and Wong et al. [38]. This includes the data of 109 patients. ^2^ Adverse effects severity data was extracted from the following articles: Gross et al. [26], Ferraroto et al. [25], Wong et al. [38], and Ascierto et al. NEO CESQ study [39], including data from 132 patients.

## Data Availability

No new data were created or analyzed in this study. Data sharing is not applicable to this article.

## Abbreviations

The following abbreviations are used in this manuscript:

SCC	Squamous cell carcinoma
BCC	Basal cell carcinoma
cSCC	Cutaneous squamous cell carcinoma
CPR	Complete pathological response
MPR	Major pathological response
PPR	Partial pathological response
NR	No pathological response
ORR	Objective response rate
TRAEs	Treatment-related adverse effects
DSS	Disease-specific survival
DFS	Disease-free survival
EFS	Event-free survival
QoL	Quality of life
ICIs	Immune checkpoint inhibitors
SOT	Solid organ transplants
NAIT	Neoadjuvant immunotherapy treatment

## Appendix A

**Table A1:** Methodological Index for Nonrandomized Studies (MINORS).

**Authors**	**Clearly Stated Aim**	**Inclusion of Consecutive Patient**	**Prospective Collection of Data**	**Endpoints Appropriate to the Aim of the Study**	**Unbiased Assessment of the Study Endpoints**	**Follow-Up Appropriate for the Aim of the Study**	**Loss to Follow-Up**	**Prospective Calculation of Study Size**	**MINORS Score**
Gross et al. [17]	2	2	2	2	2	2	1	0	13
Rischin et al. [11]	2	1	2	2	2	2	1	1	10
Gross et al. [16]	2	2	2	1	2	2	0	1	11
Ferrarotto et al. [15]	2	2	2	2	2	2	2	0	14
Ferrarotto et al. [14]	2	2	1	1	2	1	2	0	11
Kim et al. [21]	2	2	2	1	2	1	2	0	12
Godfarb et al. [23]	2	1	0	1	2	1	1	0	8
